# Vision-Based Detection of Low-Emission Sources in Suburban Areas Using Unmanned Aerial Vehicles

**DOI:** 10.3390/s23042235

**Published:** 2023-02-16

**Authors:** Marek Szczepański

**Affiliations:** Department of Data Science and Engineering, Silesian University of Technology, Akademicka 16, 44-100 Gliwice, Poland; marek.szczepanski@polsl.pl

**Keywords:** air pollution, aerial imaging, image processing, object detection, deep learning, video processing

## Abstract

The paper discusses the problem of detecting emission sources in a low buildings area using unmanned aerial vehicles. The problem was analyzed, and methods of solving it were presented. Various data acquisition scenarios and their impact on the feasibility of the task were analyzed. A method for detecting smoke objects over buildings using stationary video sequences acquired with a drone in hover with the camera in the nadir position is proposed. The method uses differential frame information from stabilized video sequences and the YOLOv7 classifier. A convolutional network classifier was used to detect the roofs of buildings, using a custom training set adapted to the type of data used. Such a solution, although quite effective, is not very practical for the end user, but it enables the automatic generation of a comprehensive training set for classifiers based on deep neural networks. The effectiveness of such a solution was tested for the latest version of the YOLOv7 classifier. The tests proved the effectiveness of the described method, both for single images and video sequences. In addition, the obtained classifier correctly recognizes objects for sequences that do not meet some of the initial assumptions, such as the angle of the camera capturing the image.

## 1. Introduction

### 1.1. Air Pollution and Its Impact on Our Health

Air pollution is a severe problem in the modern world. For example, greenhouse gas emissions affect catastrophic global climate change. According to information presented in the European Environment Agency report [1], air pollution is the leading cause of premature death and disease and is the most significant environmental health risk in Europe, being responsible for approximately 400,000 premature deaths per year in the European Economic Area. However, it is not only industrial emissions that directly affect our quality of life; the biggest impact on our health is particulate matter emissions and the smog that results. According to data available in the EEA’s annual reports on air quality in Europe, it is so-called “low emissions”, i.e., emissions arising at low altitudes, mainly from the burning of coal and other solid fuels to heat homes, that are responsible for the vast majority of particulate matter pollution. Air pollution reports usually give statistics on dust concentrations of various particle fractions: PM 10 and PM 2.5, a mixture of airborne particles with diameters smaller than 10μm m and 2.5μm, respectively. Particulate matter can contain toxic substances such as polycyclic aromatic hydrocarbons (e.g., the carcinogen benzo[a]pyrene), heavy metals, dioxins, and furans. According to The National Centre for Emissions Management (KOBiZE), emissions of pollutants from the combustion of solid fuels for the heating of residential homes are responsible for the vast majority of pollutants that are harmful to our health [2,3].

**Figure 1:**
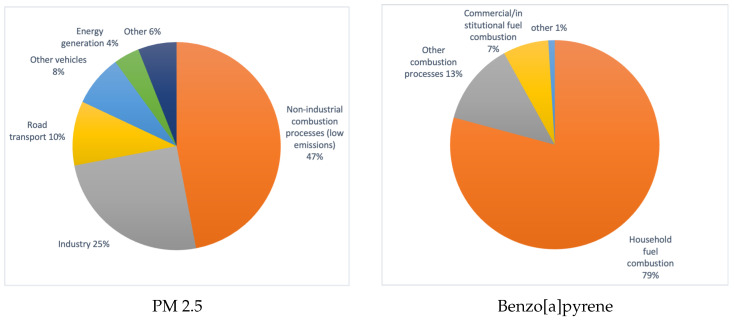
Main sources of harmful pollutants in Poland—PM2.5 and benzo[a]pyrene in 2017.

Figure 1 shows the percentage distribution of Poland’s most significant sources of PM 2.5 and Benzo[a]pyrene emissions in 2017. The charts were prepared based on data reported by KOBiZe and presented by Polish Smog Alert [2,3].

The level of particulate matter pollution is monitored both by government units, such as the Chief Inspectorate for Environmental Protection, as well as by numerous companies, non-governmental organizations, and private individuals. Comparing government data and data from other measurement networks, it is clear that the official measurements of the Chief Inspectorate of Environmental Protection, although alarming, do not fully reflect the severity of the problem. Official measuring stations are not very numerous, and they are usually located in urban areas. However, the scale of air pollution in the countryside seems to be much greater, which is confirmed by analyses of measurement data from non-governmental sources. Despite numerous programs to reduce air pollution, Poland is one of the infamous “leaders” in European and global air pollution rankings [1], mainly due to emissions of particulate matter associated with the combustion of solid fuels in residential homes. Local governments are increasingly introducing regulations restricting the use of solid fuels for heating buildings. An example of this is the anti-smog resolution for Cracow of 15 January 2016 or the resolution of the Silesian Regional Assembly of 7 April 2017. The problem of air pollution is particularly serious in southern Poland, where hard coal, often of poor quality or burned very inefficiently, has traditionally been used for years to heat households. In addition, the 2022 energy crisis has caused many ecological projects to be reversed or curtailed.

In order to effectively enforce restrictions on the combustion of solid fuels, it is necessary to monitor the actual sources of pollutant emissions effectively. Moreover, such monitoring will make it possible to verify the number of active old-type heating furnaces with the data entered in the government register of building emissions. One way to solve this problem is to use computer vision methods to detect smoke from unmanned aerial vehicles. Such a solution can take the form of scheduled regular flights to record video footage for later analysis. Analysis of the recorded material can be carried out by computer vision methods using a sufficiently powerful workstation. Another solution is a real-time implementation that indicates potential emission sources to the operator. The second solution is much more challenging to implement, but not necessarily as effective as the offline solution, because the operator can verify the correctness of the suggestions coming from the AI system. In the presented work, we will focus on the first solution; however, the proposed solution has the potential for online implementation.

### 1.2. Vision-Based Smoke Detection Techniques

Numerous solutions to the problem of smoke and fire detection can be found in the literature, but unfortunately, the vast majority of the proposed solutions relate to fire protection and forest fire detection. Most often, static cameras are used for this purpose, which continuously record image data.

#### 1.2.1. Smoke Detection from Static Cameras

As mentioned above, a large part of smoke and fire detection algorithms have been developed for fixed surveillance cameras; an extensive study of such solutions can be found in the article [4]. The majority of such methods use motion detection as a first step. The most widely used motion detection methods for smoke and fire detection are

- Determining the motion gradient using difference frames [5,6];
- Detection of chrominance changes—smoke usually has a lower chrominance value [7];
- Estimation and background subtraction using Gaussian Mixture Model (GMM) algorithms [8];
- Block-based motion estimation, similar to techniques used in modern video compression algorithms [7,9];
- Optical flow based techniques [10].

The next stage is usually the classification of areas containing smoke; due to the high variability and diversity of the phenomenon, this is not a trivial issue. Analysis of features derived from the physical properties of smoke can be useful for building a classifier. For example, areas obscured by smoke are characterized by reduced saturation, so the classification can use the relevant chromatic features for the smoke area [7]. Unfortunately, such an approach in autumn and winter conditions has very limited performance due to the generally low saturation of images acquired outdoors, which is even more apparent for aerial images. Other features based on smoke properties have been presented in the literature, which seems more versatile, e.g., local loss of focus in smoky areas or dynamic smoke properties [7,11,12,13]. Another interesting approach is the use of energy measures for areas in images based on the Discrete Wavelet Transform (DWT) [5,8] or the Discrete Cosine Transform (DCT) [9]. Another group of approaches use Local Binary Patterns (LBP) [11,12,14] and their dynamic, spatiotemporal version—Local Binary Motion Patterns (LBPM) [15]. Often, the features used for motion detection can also be used at the classification stage. An example is motion features determined by optical flow methods [6].

In recent years, deep learning techniques, especially those using convolutional neural networks, have become increasingly common, so it is not surprising that increasing numbers of such solutions are being applied to the problem of smoke detection [14,16,17,18,19,20,21].

#### 1.2.2. Smoke Detection Using Unmanned Aerial Vehicles

In recent years, the use of UAVs to monitor forest areas for early detection of wildfires has become increasingly popular; such solutions offer greater flexibility and the ability to monitor large areas. The use of drones also allows us to intensify monitoring during hot and dry weather, increasing the risk of wildfires, and it is not necessary to erect numerous observation towers; an overview of UAV-based solutions has been collected in the works [18,19]. Drone video data are usually taken on the move, especially when large areas need to be monitored. Therefore, most of the methods described above are not directly applicable.

It is challenging to find works in the literature that effectively solve the problem of smoke and fire detection with classical rule-based methods or using classical machine learning techniques; most solutions use deep learning approaches [4,18,22]. Traditional methods based on feature extraction and color transformation can be effective for fire detection [23], but their application to smoke is much more problematic. In addition, manual feature selection and engineering takes a very long time and requires domain experts to select valuable features that can make machine learning algorithms more effective.

However, most effective solutions are based on deep learning methods. The proposed approaches can be divided into several groups—those based on image classification, object detection, and semantic segmentation [19]. The first group of algorithms evaluates which class the analyzed image should be assigned to; in the case of wildfire detection, it is a matter of determining whether smoke or fire is visible in the image. Solutions using CNN architectures for UAV image classification are presented in the works of [24,25,26,27]. The architectures used to accomplish these tasks included AlexNet [28], GoogleNet [29], and VGG-Net [30].

The next group of algorithms aims to detect objects in the scene, which is more difficult because it requires finding and labeling objects in the image, usually by bounding boxes. In recent years, numerous algorithms have been proposed to solve the problem of object detection, which can be divided into two classes—two-stage and single-stage detectors. The most popular group of two-stage or region-based algorithms are R-CNN and its subsequent modifications, Fast R-CNN and Faster R-CNN [31,32]. Among the single-stage algorithms are different variants of YOLO, SSD, or Retina-Net [33]. Some solutions are based directly on visible light sensor data [18,34,35], while others also use additional sensors such as infrared or thermal imaging [36]. Another paper by Hossain et al. [22] uses additional color features and LBP together with the YOLO algorithm.

Among the most demanding are semantic segmentation algorithms; they allow, for example, to accurately distinguish areas of smoke, fire, and background. Unfortunately, they are also the most demanding in terms of computational complexity and the time required to prepare the training set. In the literature, one can find the application of various smoke and fire segmentation techniques on images and video captured with UAV platforms, for example, DeepLab [37], U-Net [38,39,40], Segnet [41], Mask R-CNN [42], or the recently introduced YOLOv7 and YOLOv8 algorithms [43].

A significant disadvantage of deep-learning methods is the need to prepare large training sets, usually labeled manually by humans. The lack of generally available UAV-based fire and smoke datasets is one of the biggest problems facing deep learning developers and researchers. This work tries to solve the problem of detecting smoke emitted by residential house chimneys. Although it appears similar to wildfire detection, this issue is a different, undeveloped problem. Smoke that should be detected is often much less visible, and the background is much more heterogeneous. When analyzing various solutions for wildfire detection, it is possible to identify some objectives and possible ways to solve our problem. Using classical machine learning methods or rule-based detection, we can effectively detect smoke in static sequences using the dynamics of smoke motion. However, we should use deep learning techniques to detect smoke effectively with a UAV platform in motion. In our case, to locate the source of the smoke, we do not need an accurate segmentation; we require a bounding box enclosing the smoke or the building emitting it.

However, as mentioned above, a large amount of input data are required to learn DL algorithms, and the lack of sufficiently large training databases is also a problem for the much better-developed wildfire detection problem. This work uses the author’s own collection of video sequences acquired between 2019 and 2022, but manually tagging this data would be very tedious and time-consuming. In addition, the areas containing smoke visible from a bird’s eye view are very diverse, and we are often unable to correctly label the areas ourselves. In such a situation, to correctly create a classifier with sufficient generalization, it is necessary to prepare even more diverse training sets than for standard detection and classification applications. Thus, it is necessary to prepare an extensive set of image data acquired in different locations, at different times of the year, times of the day, in different weather, and preferably, with different cameras.

In addition, care should be taken to ensure that the learning set includes examples with and without smoke for individual buildings; otherwise, the detector may learn to recognize buildings that usually emit pollutants rather than smoke. Thus, the work presented here proposes a mixed solution; a separate algorithm based on classical, rule-based methods will be used to create a training set that will be used to learn the actual DL detector. However, the author’s main contribution is the automation of training set preparation; the algorithm used for final detection is of secondary importance in this case. It provides a validation of the prepared training data. This paper will first present the selected UAV image acquisition plan, which, in the author’s opinion, is the most advantageous for solving the described problem (Section 2). Section 3 will present an algorithm for smoke detection in stationary video sequences. Then, in Section 4, the results of the algorithm created for stationary sequences will be used to create an extensive learning set for the YOLOv7 detector. The last section will include a summary and discussion of the results.

## 2. Detecting Smoke from Low Emission Sources in Aerial Photography

The classic solutions for static videos described in the previous section cannot be directly applied to detect low-emission smoke in aerial photographs or video sequences. The first problem is the acquisition of static sequences and the considerable visual variety of smoke emitted from household heating systems. One of the first challenges during problem analysis is the selection of the appropriate flying platform for image acquisition, the preparation of detailed assumptions regarding the method of data acquisition, and, finally, the preparation of the flight plan.

### 2.1. Choosing a UAV Platform

Acquisition of aerial imagery can, in the general case, be carried out using both manned and unmanned aircrafts. Until recently, most of the material was mainly acquired using aircraft equipped with special data acquisition kits. However, much cheaper solutions using unmanned aircraft or popular drones are increasingly being used. Several platforms on the market are dedicated precisely to photogrammetric flights, equipped with camera and sensor systems, as well as advanced positioning systems, enabling localization with centimeter accuracy—RTK (Real-Time Kinematic) and DGPS (Differential GPS).

A separate issue is the choice of aircraft type, with a choice between multi-rotor and fixed-wing platforms. The former has the advantage of the simplicity of operation and the ability to stay in hover, while fixed-wing can stay in the air for much longer durations. When developing a complete smoke detection platform, consider simple and cost-effective solutions. Thus, the study used DJI’s standard four-rotor platforms. Most of the data were acquired using DJI’s Mavic Air, Air 2s, and Mavic 2 Enterprise Dual drones—the latter is equipped with both a standard RGB camera and a thermal imaging camera. The use of thermal imaging at a later stage of the work can assist in locating active chimneys that are point sources of heat.

### 2.2. Image Acquisition—Flight Plan

Using four-rotor flying platforms, we have a great deal of freedom in choosing how to acquire data; unlike airframes, we can acquire almost static sequences by keeping the aircraft in hover. When planning a flight, we need to consider the following factors:

- Type of data acquired—video in motion, stationary video, static images (also orthophotos acquired from photogrammetric flights);
- The angle and tilt of the photos—vertical, near-vertical, tilted and, perspective;
- Acquisition band—RGB, multi/hyperspectral, and thermal imaging;
- Altitude and flight range.

The first stage of the proposed algorithm is to automatically generate a training set for the neural network. It should be as simple and efficient as possible. For this purpose, it is best to use stationary sequences. It is possible to acquire “almost static” sequences thanks to good stabilization of the drone’s position and additional mechanical stabilization of the camera. For the later stages of the study, photogrammetric flights were also carried out, resulting in a series of images that enabled the creation of an orthophoto map. It was decided that the image would be captured with a camera pointing vertically downward (nadir). Due to hardware limitations, most of the data were acquired using only the RGB sensor, but thermal imaging is also available for some of the data.

A distinct problem is the choice of flight altitude and camera focal length. A flight altitude that is too low limits the acquisition area, while one that is too high can prevent correct detection. When preparing the flight plan, it is necessary to consider the currently applicable airspace restrictions. At the initial stage of the project, following the legal restrictions in place at the time, a wide range of flight altitudes was considered, but the legislative changes introduced at the beginning of 2021 in the EU limited the maximum flight altitude to 120 m.

**Figure 2:**
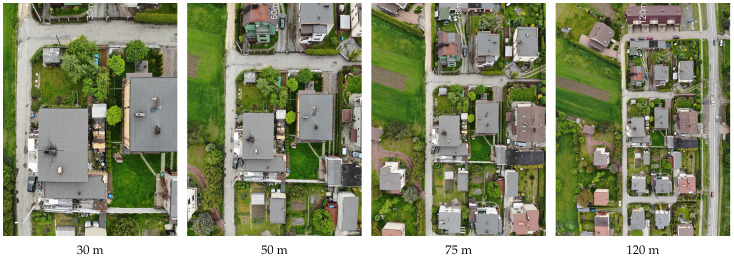
Example frames captured from a drone for different altitudes.

Figure 2 shows sample images obtained for different flight heights—a ceiling that is too low clearly limits the detection area, while at the same time, it can pose a privacy problem for residents. Therefore, it was decided to choose the highest possible ceiling—that is, 100 or 120 m (depending on the airspace restrictions for the area).

## 3. Smoke Detection Algorithm in Stationary Video Sequences

**Figure 3:**
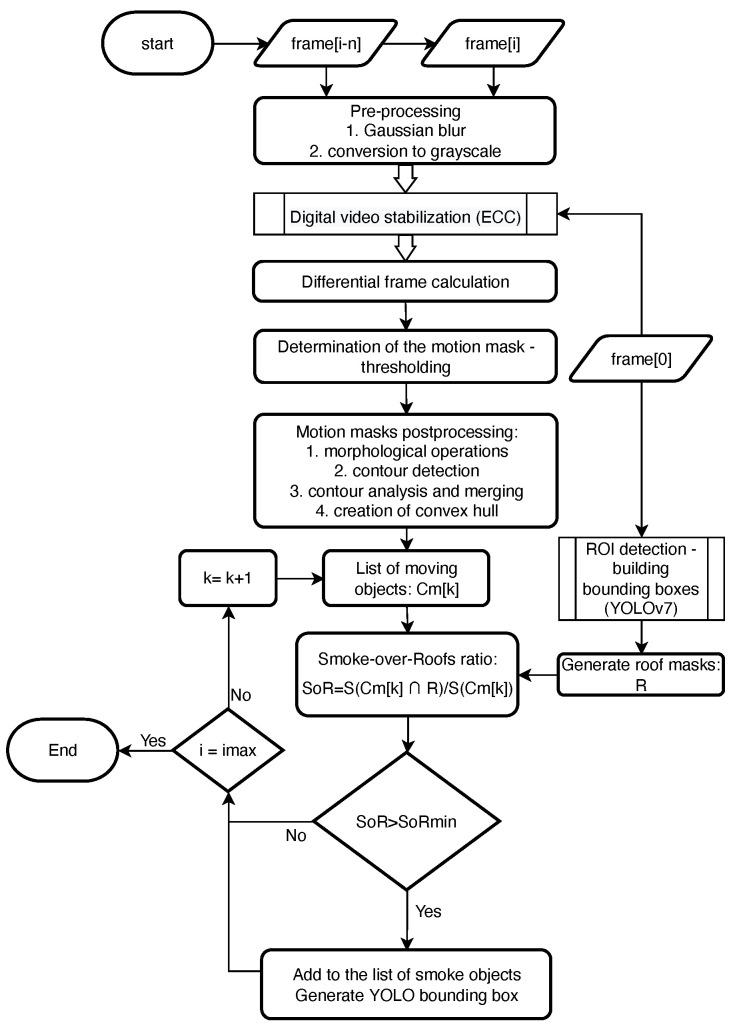
Block diagram of smoke detection algorithm in stationary video sequences.

The acquisition of static video sequences requires the preparation of a complex flight plan and takes a lot of time—the drone must remain hovering for about 10 s at each measurement point. The primary purpose of the solution used is to prepare a sufficiently large training set with automatically labeled smoke regions; by design, this part of the algorithm is to work offline. The proposed algorithm for smoke detection in stationary sequences can be divided into the stages shown in the block diagram (Figure 3).

Subsequent video frames are subjected to preprocessing and stabilization. Next, the masks of moving objects are determined on the basis of the differential frames. In the next step, the obtained masks are subjected to a series of operations to determine consistent objects with convex contours. Finally, we check which moving objects overlap the roof areas; we assume that such objects define the smoke area. Roof areas were determined in a separate process using the YOLOv7 classifier [43].

### 3.1. Motion Masks Detection

#### 3.1.1. Preprocessing and Digital Video Stabilization

The differential frame motion detection method assumes that image sequences are acquired with a stationary camera. Unfortunately, for the natural sequences acquired from the multi-rotor drone, this condition is not fulfilled—despite the effective stabilization of the position and the camera itself, subtle image shifts are still visible. These shifts in some situations, such as during strong winds, are large enough to interfere with other motion and background detection algorithms, such as MOG background subtractor, as well [8]. The problem stems from the fact that the rate of change in the image resulting from the drone’s movement has similar dynamics to the objects being detected—namely, smoke. It is necessary to use another method to eliminate the impact of small camera displacements; this solution proposes to stabilize video frames using the Enhanced Correlation Coefficient Maximization (ECC) algorithm [44]. The work presented here assumes the Euclidean model of displacement and assumes that successive frames are aligned to the initial one. Such an assumption remains correct for relatively short sequences; however, in our case, this condition is met—the length of the test sequences was only 10 s. In addition, Gaussian blurring, with σ=1.4, was used to cancel out noise and reduce the impact of moving small elements with distinct edges (such as branches).

#### 3.1.2. Motion Masks from Gradients

The objective of the presented work is to provide an efficient smoke detection algorithm that is as simple as possible. It was decided to determine the time gradient of the frames after additional stabilization. The smoke is characterized by relatively small movement dynamics; even in fairly strong winds, the differences between successive frames are barely visible, so it is necessary to increase the time window size *n* for which the gradient is determined (in this work, the value of n=10 is assumed). Since in most input sequences, the smoke is characterized by low saturation and the most temporal variation occurs in the luminance channel, we will determine the differences between frames only for luminance:

(1) $$Y\left(i\right)=0.299\cdot F_{R}\left(i\right)+0.587\cdot F_{G}\left(i\right)+0.114\cdot F_{B}\left(i\right),$$

where $F_{R}$, $F_{G}$, and $F_{B}$ are RGB components of frame F(i), while *i* denotes frame number. A simple frame subtraction operation was then applied to determine the motion gradient:

(2) $$G\left(i\right)=\left|Y(i)+Y(i-n)\right|.$$

Finally, the difference images were binarized using adaptive thresholding based on Gaussian kernel. For each frame, we get a raw mask in the form of

(3) $$M\left(x,y\right)=\left\{\begin{array}{ccc} 1, & \mathrm{if} & G(x,y)⩾G(x,y,\sigma )-c\\ 0, & \mathrm{if} & G(x,y)<G(x,y,\sigma )-c \end{array}\right.,\;$$

where G(x,y,σ) is Gaussian smoothing operator with σ=3.5, and c=4 is a additional threshold shift.

#### 3.1.3. Motion Masks Postprocessing

Motion masks obtained in this way often have irregular shapes and usually do not form coherent objects. In the presented work, it was decided to perform an appropriate combination of morphological operations and merging of close objects. At first, in order to filter out noise, an erosion with a disk-shaped structuring element of size r=2 was carried out, followed by a double dilation with the same kernel. Finally, a morphological closing with a very large structuring element of radius r=50 was carried out:

(4) $$M_{m}\left(i\right)=\left(\left(\left(M\left(i\right)⊖Disk_{r=2}\right)⊕Disk_{r=2}\right)⊕Disk_{r=2}\right)⊙Disk_{r=50}.$$

The masks created in this way can still be inconsistent and have irregular shapes. In order to reduce the number of small objects detected, and in particular, to reduce the problem of smoke object fragmentation, further processing of motion masks was carried out; Algorithm 1 depicts post-processing of mask contours:
**Algorithm 1** Motion objects contour processing**Input:**$M_{m}$—binary mask of objects in motion, h,w—height and width of the image**Output:**$C_{m}$—list of final objects in motion1:**function**filterMotionMasks ($M_{m}$)2:     min←h∗w∗0.0001                  ▹ min. contour area3:     max←h∗w∗0.2                     ▹ max. contour area4:     maxdist←h∗0.05              ▹ min. distance for contour merging5:     cnt← findContours($M_{m}$)        ▹ motion mask contours and object labeling6:     cnt← filterContoursByArea(cnt,min,max)  ▹ filtering objects of extreme sizes7:     cnt← mergeCloseContours(cnt, maxdist)      ▹ merging close objects using Euclidean distance8:     $C_{m}\leftarrow \left[\right]$9:     **for** c∈cnt **do**10:           c←convexHull(c)                  ▹ convex hulls of objects11:           $C_{m}$.append(c)12:     **end for**13:     **return $C_{m}$**                       ▹ filtered moving objects14:**end function**

**Figure 4:**
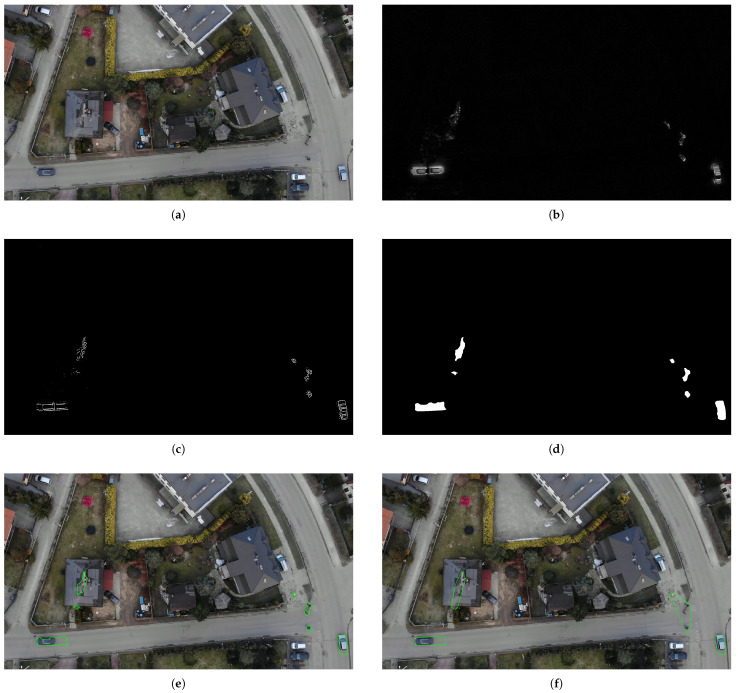

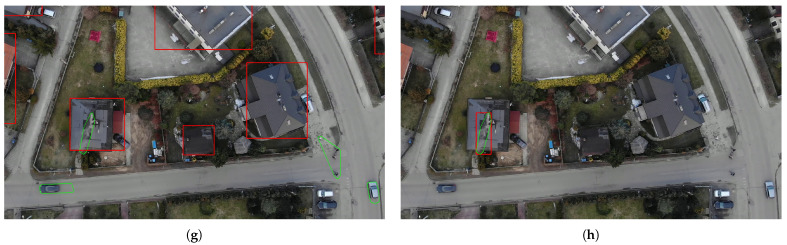
Motion mask generation (**a**–**f**) and final smoke region detection (**g**,**h**). (**a**) Input frame *F*(*i*); (**b**) Temporal gradient G(i); (**c**) Raw thresholding result M(i); (**d**) Motion masks after morphological processing $M_{m}\left(i\right)$; (**e**) Contours of objects in motion; (**f**) Final result after contour filtering; (**g**) Moving objects $C_{m}$ and rooftops *R*; (**h**) Final smoke areas.

The results of the subsequent stages of moving object detection for an example frame from the test video sequence are shown in Figure 4a–g.

### 3.2. Rooftop Area Detection

The motion mask detection process described above effectively detects most objects in motion, including slow-moving smoke objects. However, it is necessary to distinguish which masks represent smoke and other objects, such as cars, pedestrians, branches in the wind, and many others. Attempts were made to prepare a classical classifier based on the visual features of the smoke and its gradient, but despite considerable effectiveness, some objects, such as branches and flags in the wind, caused problems. It was therefore decided to take a completely different approach; given that we are looking for smoke emitted from residential chimneys, it is logical that their areas overlap, at least in part, with the areas of roofs.

#### Training Set for Rooftop Detection

It was necessary to use an effective tool for detection and, preferably, segmentation of the roof area. For this purpose, a dataset of aerial photographs from photogrammetric flights and fragments of moving video sequences was prepared for the area of southern Poland, mainly the area of the Silesian Voivodeship.

It was decided to use deep learning techniques because of the wide variety of objects to be detected, namely roofs. Unfortunately, initial attempts at accurate rooftop area segmentation resulted in numerous artifacts, resulting from, among others, substantial similarities between roofs covered with bitumen roofing felt, especially in row houses, and asphalt on the road. Due to the simplicity of labeling objects, the speed of the network’s learning, and the fact that most buildings are rectangular, it was finally decided to detect bounding boxes with the YOLOv7 algorithm.

**Figure 5:**
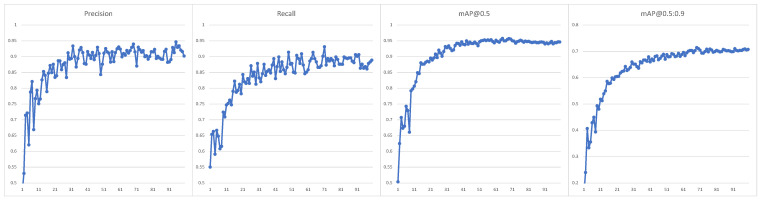
Learning process for rooftops training set.

A collection of more than 1000 images extracted from several hundred aerial photographs was prepared and tagged to train the neural network. Images were acquired in different weather conditions, times of day, and seasons of the year. Several different cameras were used and different source data were selected (still images and frames extracted from 4K videos). Most images used are in central projection, but the dataset also includes tiles from orthophotos acquired with the drones used in this project. In addition, some images have been divided into different-sized tiles to facilitate annotations and improve the learning process. The dataset was divided into a training set, a validation set, and a test set in an 8:1:1 ratio. The YOLOv7-X model and transfer learning from the MS COCO dataset [45] were used to train the network. Figure 5 shows the progress of network learning for a set of rooftops; it is clear that after 100 epochs, we get stable and acceptable results. The tagged dataset can be downloaded from location: https://tiny.pl/w4gdj (accessed on 30 December 2022).

**Figure 6:**
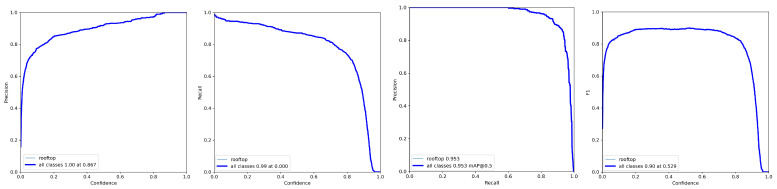
Results obtained for the testing set.

**Figure 7:**
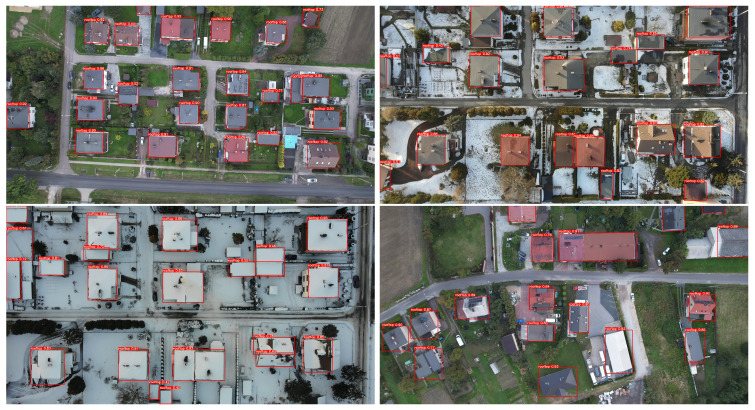
Results of the roof detection algorithm under different weather conditions.

Figure 6 shows the quality metrics obtained for the test set, while Figure 7 shows examples of building roof detection for data from the target 4K video sequences.

## 4. Final Smoke Detection and Smoke Training Set

The previous paragraphs described the process of detecting moving objects and how to detect rooftops. However, our goal is to generate a learning set containing smoke that occurs above buildings. This is due to the assumption that we want to detect sources of smoke emitted by residential furnaces and not, for example, bonfires. Thus, it can be assumed that such objects will at least partially be detected in the roof area. At the same time, it can be assumed that no other moving objects will be detected over the rooftops. Let us define, for each moving object $C_{m}\left[k\right]$, its intersection with the roof mask *R*, and then, the smoke-over-roofs (SoR) ratio, which is the relation of the intersection product area compared to the area of the entire smoke object:

(5) $$SoR\left[k\right]=\frac{S(C_{m}\left[k\right]\cap R)}{S(C_{m}\left[k\right])}.$$

We consider a given object to be smoke if the value of SoR[k]>0.1. Final results with roofs and bounding boxes used in YOLO training set generation are presented in Figure 4g,h.

### Training the YOLO Smoke Detector

A total of 542 video sequences acquired from the drones described earlier were used to create the stationary training set. The vast majority of the sequences were recorded at 4K@30fps resolution, each about 10 s in length. The data were acquired at different times of the day, year, and in varying weather. However, due to the nature of climate change in our region, sequences with snow are underrepresented. The set of video sequences used in this work is available for download from the location: https://tiny.pl/w4mx5 (accessed on 30 December 2022).

As a result of the algorithm proposed above, a set of data automatically tagged with smoke was created. During the algorithm’s operation, a maximum of every tenth frame of the sequence was saved, but the frame was skipped when no moving objects were detected. This was to limit the situation in which the training set would contain frames with barely visible smoke, but the detection would be unsuccessful (a human observer is also unable to see the smoke in all frames). On the other hand, it is possible to add an operation of averaging areas over time which would enable smoother smoke frame tagging. However, this approach may lead to a situation where the network learns specific types of roofs, and not a smoke pattern. The result was a dataset containing 17,859 frames, of which 16,591 were tagged with objects considered to be smoke. Of course, some of the tags must be wrong, but it was assumed that such data are sufficient for the correct operation of the algorithm. As in the case of roof detection, the dataset was divided into training, validation, and test sets in the ratio of 8:1:1.

**Figure 8:**
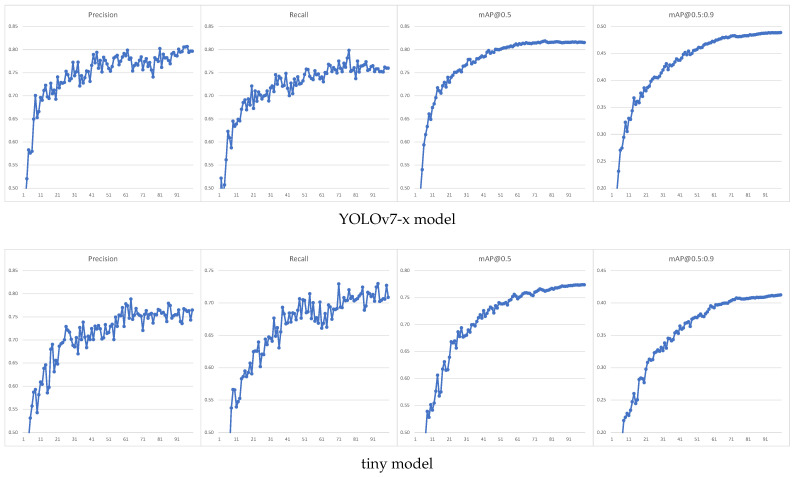
Learning process for validation set using models of different complexity.

The YOLOv7 architecture was tested for two different models: YOLOv7-x and YOLOv7-tiny. The first was the maximum size model for which we were able to obtain results in a reasonable time, and the second is the simplest model, enabling the fastest detector operation and use on mobile devices. Despite the use of 4K resolution images, we were forced to choose an image size of 1280 px in the learning process due to hardware limitations. In addition, random image scaling was used for the ’tiny’ model to improve the detection of small areas. A comparison of the training process for both models is shown in Figure 8.

**Figure 9:**
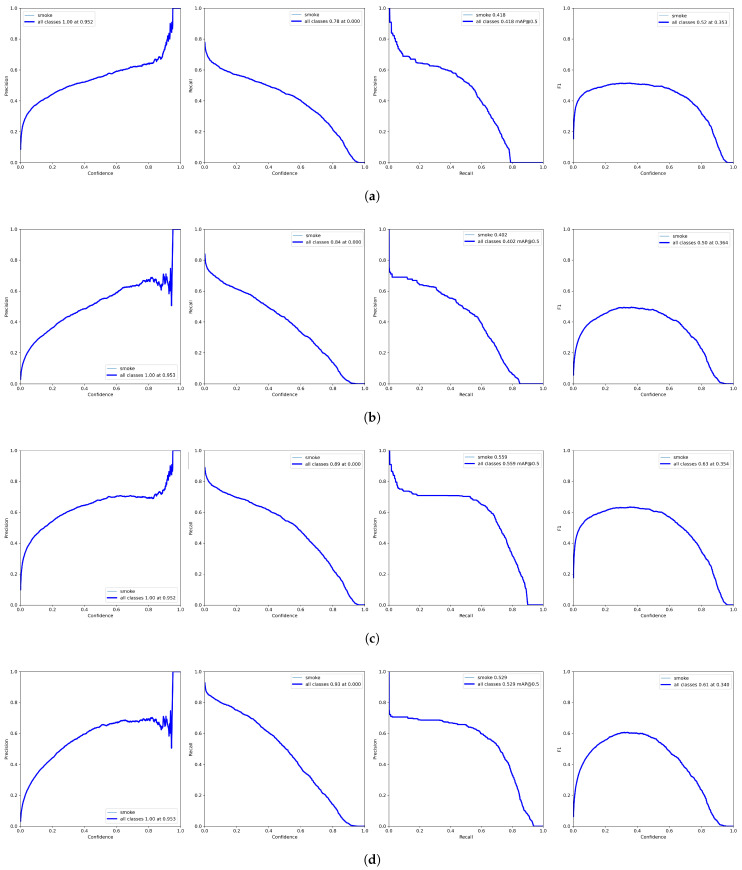
Smoke detection efficiency on testing set with different YOLOv7 models and IoU values. (**a**) YOLOv7-x model (IoU=0.5); (**b**) YOLOv7-tiny model (IoU=0.5); (**c**) YOLOv7-x model (IoU=0.1); (**d**) YOLOv7-tiny model (IoU=0.1).

As can be seen, a larger model gives better results for the validation set, but it should be remembered that this set was created in a similar way as the training set, and it may contain similar errors and is obtained based on the same test sequences; it was decided to test the classifier’s performance on a more reliable test dataset. The performance of the obtained classifiers was finally tested on a manually labeled set of images acquired at the most similar time and location as the training set. The images of the test set were captured as JPG photographs of at least 12 MP in size, with an aspect ratio of 4:3. Significantly worse results were obtained than for the automatically tagged validation set; the results for both models tested are shown in Table 1:

Detailed detector quality metrics for both models are shown in Figure 9a and Figure 9b, respectively. As one can see, for the test set, the results for both models are already comparable. The mAP@0.5 indicators, especially mAP@.5:.95, can be disappointing. However, it is important to remember what type of objects we are trying to detect. The boundaries of the smoke area are complicated to determine; in addition, for a human observer, obtaining the IoU (Intersection over Union) index at the level of 0.95 is practically impossible (hence, leading to such a low value of the mAP@0.5:0.95 index). Even obtaining a value of 0.5 can be difficult. In fact, we want the algorithm to correctly recognize smoke sources and correctly determine their area, which is why we also analyzed the detection quality indicators for the value IoU=0.1 (Figure 9c,d).

**Figure 10:**
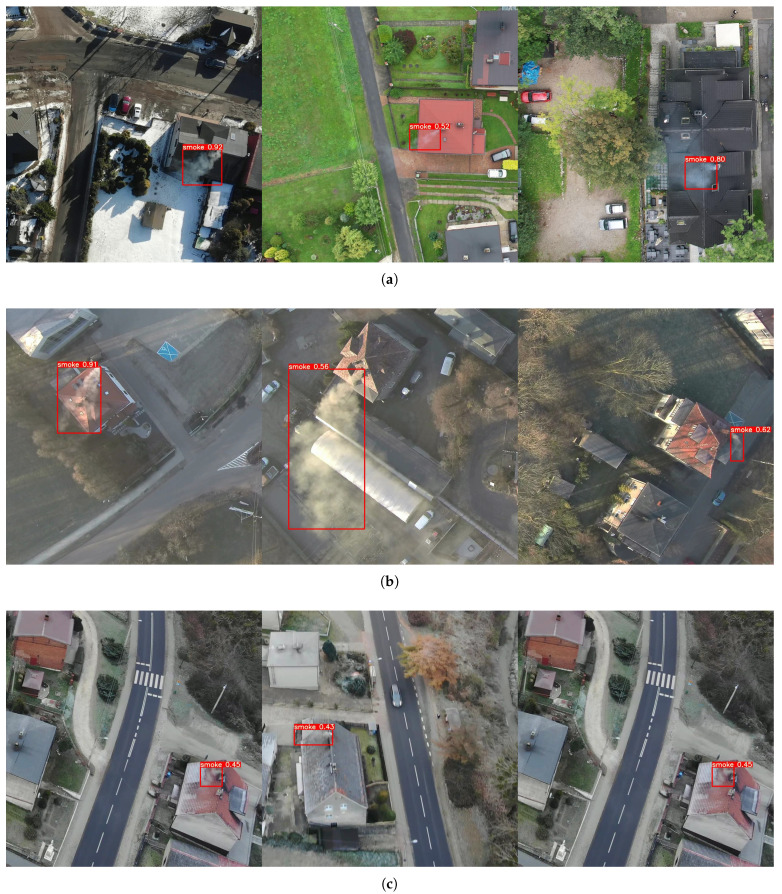
Example detection results for the YOLOv7-x model. (**a**) Detections from DJI Drones; (**b**) Detections from Xiaomi Mi Drone Video; (**c**) Detections for oblique flight.

In addition to synthetic tests, an evaluation of the detectors’ real-world performance for video sequences was also carried out; it turns out that the detection efficiency for the sequence is significantly higher—the detector finds almost all sources of smoke, at least on some of the tested frames. Figure 10a shows detection from DJI drone flights. The results from a tough case are presented on Figure 10b. This sequence was acquired with a completely different device—(Xiaomi Mi Drone 4K), and the flight was carried out for an area not represented in the training set. During the tests, it was noticed that the algorithm is also quite effective for oblique flight; however, to increase the universality of the detector for such a view, it is necessary to extend the training set appropriately (Figure 10c).

## 5. Summary and Future Works

The presented work describes the problem of detecting smoke resulting from so-called low emissions. This problem is not common in the literature, even though it is important for human health and environmental protection. The paper uses some ideas known from wildfire detection approaches, but the peculiarities of the problem under study prevent direct implementation of the mentioned techniques.

A multi-step algorithm was proposed to solve the problem. The first part, designed to work offline, requires a well-planned and executed measurement experiment. It was necessary to acquire stationary aerial image sequences for smoke-emitting buildings. Such sequences were recorded for different locations, times of day, and seasons of the year, under different weather conditions.

For the data prepared in this way, an algorithm for detecting moving objects was created. Whether such an object should be treated as smoke generated in the building was made based on an assessment of the object’s location in relation to the buildings. The area of buildings was determined by a properly prepared classifier using YOLOv7 architecture.

However, such a solution has some drawbacks, and buildings are marked as bounding boxes rather than exact outlines. This can cause objects moving near buildings to be incorrectly classified as smoke in certain situations, such as the oblique location of buildings relative to the image axis. This results in the introduction of erroneous samples into the training set generated, resulting in poorer performance of the final smoke detector. This problem can be reduced by adding a stage of smoke classification using its visual properties. We are working on applying a classification stage based on the Haralick’s GLCM features [46] and energy measures, such as entropy, determined in both the image and gradient domains. Preliminary results using the Light GBM classifier [47] show promising results (close to 95% efficiency for correct binary object classification for the test set). However, creating the final training set will verify these results. Another idea being tested is to use segmentation using classic machine learning algorithms to better segment differential images. It seems that such improvements will allow a significant increase in detector efficiency.

The first stage of the algorithm can be used independently for the effective detection of smoke objects, but its practical application can be cumbersome; in our case, it was used as an intermediate stage to automatically generate a training set for a convolutional neural network. The created collection was used to learn the YOLOv7 network. Its effectiveness was then demonstrated for network models of different complexity. It has been found that even the simplest YOLOv7-tiny model enables effective smoke detection from high-resolution video sequences.

This allows the implementation of smoke detection also on mobile devices; it would be possible to display appropriate notifications to the drone operator: this could occur when using an advanced device with a sensor to measure the composition of the smoke. The described algorithm, with minor modifications, could also be used to detect other sources of smoke, including fires.

In this work, we focused on the first stage of the algorithm, using ready-made YOLOv7 models with default parameters. However, we can improve final classifier efficiency, not only by improving the quality of the training set generated, but also by optimizing the model and parameters of the neural network, or even by choosing different architectures.

There are plans to further expand the set of video sequences: particularly, to include sequences in oblique view. This will make it possible to improve detection performance for such a camera view, which is advantageous because we have a larger field of view, and the smoke is usually more visible in this view.

## Figures and Tables

**Table 1:** Smoke detection efficiency for the test set for different sizes of YOLOv7 models.

	P	R	mAP@.5	mAP@.5:.95
YOLOv7-x	0.513	0.518	0.418	0.236
YOLOv7-tiny	0.472	0.521	0.402	0.213

## Data Availability

The data used in the paper is available for download from the article author’s repository from the locations: https://tiny.pl/w4gdj and https://tiny.pl/w4mx5, (all accessed on 30 December 2022).

## Abbreviations

The following abbreviations are used in this manuscript:

ECC	Enhanced Correlation Coefficient Maximization
EEA	European Environment Agency
DCT	Discrete Cosine Transform
DWT	Discrete Wavelet Transform
DGPS	Diferential GPS
GPS	Global Positioning System
IoU	Intersection over Union
KOBiZE	Krajowy Ośrodek Bilansowania i Zarządzania Emisjami
	—The National Centre for Emissions Management
LBP	Local Binary Patterns
MOG	Mixture of Gaussian
MP	megapixel
RTK	Real-Time Kinematics
SoR	Smoke-over-Roofs ratio
UAV	Unmanned Aerial Vehicle
